# Convergence of per capita ecological footprint among BRICS-T countries: evidence from Fourier unit root test

**DOI:** 10.1007/s11356-023-26532-1

**Published:** 2023-03-23

**Authors:** Yuksel Bayraktar, Kenan Koc, Metin Toprak, Ayfer Ozyılmaz, Mehmet Firat Olgun, Daniel Balsalobre-Lorente, Ozgur Bayram Soylu

**Affiliations:** 1 Department of Economics, Ankara University, Ankara, 06500 Turkey; 2 Department of Economics, Mus Alparslan University, Muş, 49250 Turkey; 3 Department of Economics, Istanbul Sabahattin Zaim University, Istanbul, 34303 Turkey; 4 Department of Foreign Trade, Kocaeli University, Kocaeli, 41650 Turkey; 5 Technology Transfer Office, Kastamonu University, Kastamonu, 37150 Turkey; 6 University of Castilla-La Mancha, Ciudad Real, Spain; 7 Department of Economics, Kocaeli University, Kocaeli, Turkey

**Keywords:** Ecological footprint, Convergence, Unit root test, BRICS-T

## Abstract

In recent years there has been a great deal of research into environmental pollution using a variety of techniques in response to growing environmental concerns. Convergence analysis, one of these techniques, helps determine whether the developing countries will catch up with the rich countries in pollution using unit root tests. However, the vast majority of the research in the field has generally used conventional unit root tests. Since many economic series contain structural breaks, using unit root tests that account for structural breaks is essential for accurate prediction. More specifically, if the series has a fractional process, conventional unit root tests may erroneously conclude that the departure from linearity is permanent. Moreover, the existing literature mainly uses gas emissions, such as carbon dioxide, which represent pollution weakly. Therefore, we use per capita ecological footprint (EF hereafter) as a more comprehensive pollution indicator of environmental degradation. In this direction, the study aims to determine whether BRICS-T countries' EF converges to the average of the BRICS-T for the 1992–2017 period. Besides the ADF unit root test, we employed the Fourier ADF unit root test, which considers the structural breaks, and the Fractional Frequency Fourier ADF unit root test, which accounts for structural breaks by considering fractional values. Our results showed that EF converges in Russia and Turkey according to the conventional ADF test, in China and Russia according to the Fourier ADF test, and in Brazil and China according to the Fractional Fourier Frequency test.

## Introduction

Environmental problems such as climate change and global warming, described as different dimensions of environmental pollution, negatively affect the world ecosystem in various aspects. A critical factor in the emergence of these problems is the increase in greenhouse gas emissions in the atmosphere. With the development of industrialization, the demand for energy increases, mainly met by fossil fuels such as coal, natural gas, and oil. This leads to an increase in greenhouse gas (GHG) emissions. Therefore, numerous meetings and conferences have been organized to prevent the growth of GHG emissions and, thus, global warming. The Kyoto Protocol is one of the most recognized multinational agreements to control GHG emissions (Burnett 2016). The GHG emissions consist of six different gas types as classified in the Kyoto Protocol, which are carbon dioxide (CO_2_), methane gas (CH4), nitrogen oxide (N_2_O), hydrofluorocarbons (HFCs), perfluorocarbons (PFCs), and sulfur hexafluoride (SF) (Ahmed et al. 2017). However, as there are several pollution dimensions, an assessment of pollution based on these gases alone might be a handicap. In addition, the Kyoto Protocol sets greenhouse gas reduction targets for industrialized countries, ignoring developing ones (Erdogan and Okumus 2021). This could be a further drawback, as developing countries are responsible for the vast proportion of GHG emissions.

Since CO2 emission accounts for the largest share of GHG emissions, most studies focus on it, which has led to a significant imbalance in the environmental pollution convergence literature (Ozcan et al. 2019; Williams III 2016). Although the increasing trend of CO_2_ emissions has been reduced thanks to technological developments and regulatory policies, the consideration of CO_2_ emission as an environmental quality indicator has been criticized due to the increasing trend of other pollutants (Stern 2017). In parallel with this problem, countries’ efforts to control pollution by focusing on CO_2_ emissions lead policymakers to take inappropriate measures that expose the countries to undesirable outcomes. For example, decreasing gases may mislead policymakers to relax the standards, thinking that pollution is improving despite increased water pollution, deforestation, and declining diversity. On the other hand, EF is a more comprehensive indicator of environmental degradation, and it already includes CO_2_ emission under its “carbon field” component. In addition to the carbon field, EF includes five other components: built-up field, grassland field, fishing field, forest field, and agricultural field (Wright et al. 2011). EF is a better indicator of environmental quality or sustainability than CO_2_ emission (Haider and Akram 2019).

The theoretical underpinning of the convergence of environmental quality is based on the catch-up hypothesis, which draws from the environmental Kuznets curve (EKC) proposed by Brock and Taylor (2003). According to the Environmental catch-up hypothesis, the gap between the environmental quality in rich and developing countries will narrow as developing countries begin to use environmental technologies as their income levels rise, and environmental pollution indicators will converge (Yilanci and Pata 2020). Therefore, studies on pollution are generally discussed within the EKC (Ahmed et al. 2017; Balsalobre-Lorente et al. 2022). The EKC is named so because of its similarity to the curve known as the Kuznets curve in the literature, which was put forward by Simon Kuznets, who studied the relationship between economic growth and income distribution in the 1950s (Ulucak and Bilgili 2018). The Kuznets curve suggests that income inequality increases at the beginning of economic development in a country, but this inequality decreases after a certain income level (Kuznets 1955). In the following periods, this relationship was adapted to the relationship between the environment and the economy. According to the EKC, while environmental degradation occurs with increased income, it tends to decrease after a certain income level (Diallo 2004). The EKC was first addressed by Grossman and Krueger (1991) to examine the effects of the North American Free Trade Agreement (NAFTA) on the environment. The study found a positive relationship between income per capita and environmental pollution at low-income levels in 42 countries. In contrast, this relationship was found to be negative at high-income levels. After this pioneering study, most research in the literature employs CO_2_ emissions within the EKC models (Ulucak and Bilgili 2018).

Environmental convergence has gained considerable popularity in the academic literature thanks to its relevance to Sustainable Development Goals. In essence, the concept seeks to explore whether environmental policies across countries tend to become more similar over time (Pradhan et al. 2017). To test for convergence, researchers typically use unit root tests. Since many economic series contain structural breaks, tests that account for structural breaks improve the accuracy of the results. The test developed by Enders and Lee (2012) takes structural breaks into account. The fractional Frequency Fourier ADF unit root test developed by Bozoklu et al. (2020) accounts for structural breaks considering fractional values to determine the optimal lag length. However, most existing studies do not consider fractional integration with structural breaks, but there are rational reasons for the fractional integration of the series. If series have a fractional process, conventional unit root tests may lead to the erroneous conclusion that the departure from linearity is permanent. The fractional integration approach, therefore, provides a more general framework for examining whether there is evidence of persistence and non-stationarity in the series (Bozoklu et al. 2020).

The motivation of this paper lies in three points: (i) the vast majority of the existing literature does not consider fractional integration with structural breaks, (ii) furthermore, the available literature mainly uses GHG emissions as a pollution indicator, and (iii) as far we know, no study is investigating BRICS-T countries in this context so far.

The contributions of this study are as follows (i) Firstly, because many economic series contain structural breaks in the long run, the conventional unit root tests that cannot take into structural breaks might give a biased result. We use the Fourier ADF unit root test developed by Enders and Lee (2012), which takes structural breaks into account to overcome this problem. However, this test does not consider fractional values in determining the optimum lag length. Therefore, we use the Fourier Fractional Frequency ADF unit root test developed by Bozoklu et al. (2020), which accounts for structural breaks by considering fractional values; (ii) Secondly, the existing literature mainly uses GHG emissions, particularly CO2, as an indicator of environmental pollution. But we use EF, a more comprehensive indicator that already includes CO2 as one of the six components; and (iii) Finally, since the main objective of international agreements is to minimize pollution effects across the world, investigating convergence in BRICS-T, which has an essential position in the world in terms of population, economy, and the geographical area it covers, can help the world’s pollution reduction. BRICS-T countries account for approximately 40% of the population, 26% of the GDP, and 26% of the terrestrial area. Moreover, convergence analysis of developing countries rather than developed ones would be more appropriate since EKC implies that countries pollute the environment while developing.

The remainder of this study is as follows. “Ecological footprint, biocapacity, and BRICS-T countries” section discusses the EF of the BRICS-T countries after the introduction. In “EF and convergence theory” section, convergence theory is examined within the scope of EF. In “Literature review” section, the literature is discussed. In “Data set and method” section, data and methods are presented. Finally, in “Empirical methodology” section, the analysis findings are discussed.

## Ecological footprint, biocapacity, and BRICS-T countries

EF refers to the biologically productive land and water areas that populations need to produce renewable resources and ecological services. On the other hand, biocapacity indicates a country, region, or world’s renewable resource and ecological service capacity (Galli et al. 2014). These areas, which constitute the supply part of nature and are expressed as biocapacity (Ewing et al. 2010; Monfreda et al. 2004; Wackernagel et al. 2002), are essential for basic needs. In this direction, energy for heating, fabric for clothes, wood for shelter, food for a healthy life, and ecological assets for the absorption of polluting wastes are provided by nature (Mathis and David 1998). However, since these fertile land and sea areas provided by nature are not unlimited, human demand puts pressure on the environment.

The EF is an important indicator that measures people’s demand for the ecosphere and compares consumption with the regenerative capacity of natural resources. While many EF calculation methods exist, the “national footprint” method is the most widely used. These calculations are made with data obtained from the data banks of organizations such as the United Nations Food and Agriculture Organization (FAO), the International Energy Agency (IEA), and the United Nations Statistics Division (UNSD) (Kitzes et al. 2009). National footprint calculations measure EF and biocapacity in global hectares (gha). The global hectare is the conversion of hectares to the average global bio-productivity for a given year (Ewing et al. 2010; Galli et al. 2007; Kitzes et al. 2009; Ma and Jiao 2023).

The consumption-based EF used for the analysis is obtained by adding the embedded footprint to imports to the production-based EF formed in a particular year in a country and subtracting the embedded footprint to export (Borucke et al. 2013). In Eq. (1), EFC, EFP, EFI, and EFE represent consumption, production, import, and export-based EF, respectively (Galli et al. 2014; Lin et al. 2018).

1 $${\mathrm{EF}}_{C}={\mathrm{EF}}_{P}+{\mathrm{EF}}_{I}-{\mathrm{EF}}_{E}$$

**Fig. 1:**
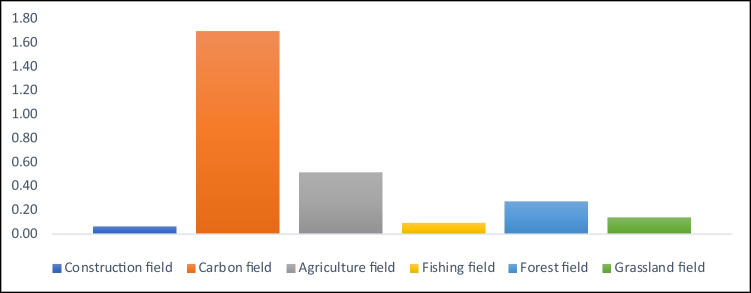
The components of ecological footprint. Source: https://data.footprintnetwork.org/#/countryTrends?type=BCpc,EFCpc&cn=5001

EF consists of six different components: agricultural field, grassland field, fishing field, construction field, forest field, and carbon field (Kitzes et al. 2007). Figure 1 shows the weights of these components in the EF. As of 2017, it is observed that the footprints with the largest share in the EF are, respectively, in the carbon field, agriculture field, forest field, grassland field, fishing field, and construction field.

As seen in Fig. 1, the EF reflects the destruction of human beings to the environment in terms of different parameters such as construction, agriculture, grassland, and fishing and excluding air pollution. Although the pressure of the carbon field on environmental pollution is relatively high, the effects of the other five components are undeniable. This process is a fundamental reason for considering the study’s EF as an environmental pollution indicator. In addition, EF gives an idea about the pressure of the population on nature and economic factors.

**Fig. 2:**
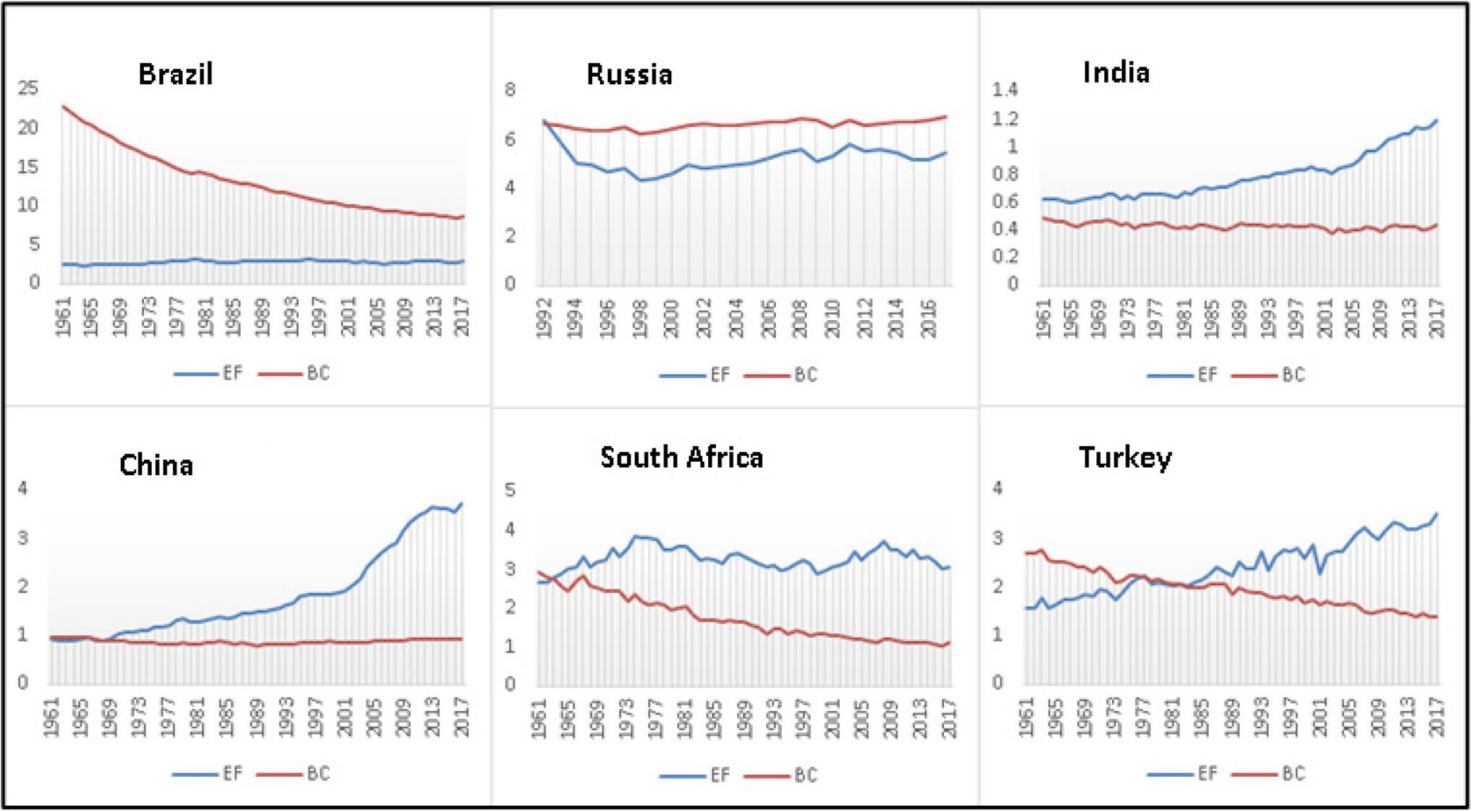
Ecological footprint and biocapacity (gha) in BRICS-T countries. Source: Global Footprint Network (2022)

Figure 2 shows the changes in the BRICS-T countries’ per capita EF and biocapacity between 1961 and 2017. It is observed that biocapacity decreases continuously in Brazil, India, South Africa, and Turkey. There was a partial change in China and Russia. Therefore, the EF tends to increase constantly, albeit in different sizes for all countries studied. If the EF exceeds the biological capacity, it is expressed as an ecological deficit; if it is below the capacity, it is defined as an ecological surplus. Accordingly, while there is an ecological surplus in Brazil and Russia, there is an ecological deficit in other countries.

While there is a tendency toward an ecological deficit in countries with an ecological surplus, the spread is narrowing. In countries with an ecological debt, the deficit is increasing, and the spread is widening. Therefore, the fact that BRICS-T countries have an important position at the global level in terms of economy, population, and area covered. The EF is a comprehensive indicator of environmental pollution convergence. This is the prominent element of the study in terms of the gap to be filled and the points that it diverges from the literature.

## EF and convergence theory

Convergence theory is based on the convergence analysis of growth dynamics, which Solow (1956) discussed within the framework of the Neo-Classical Growth Model. Convergence analysis, which has improved theoretically and empirically over time, assumes that developing countries can catch up with rich countries (Ulucak et al. 2020). Convergence occurs when a gap exists between countries’ per capita incomes, as the Neo-Classical Growth Model predicted. However, this situation, which divides the countries into different clusters, disappears as the savings, population growth, and technological development rates become similar over time. Convergence theory is classified as absolute convergence, conditional convergence, and club convergence by Galor (1996). Absolute convergence includes income convergence of countries according to their initial conditions, while conditional convergence deals with the structural similarities of countries. Club convergence includes initial conditions and structural features (Ulucak et al. 2020). Barro and Sala-i-Martin (1992) pioneered the widespread use of convergence studies in macroeconomics. Barro and Sala-i Martin analyzed whether per capita income and output converged in 48 US states.

On the other hand, environmental convergence has found its place in the academic literature in line with achieving Sustainable Development Goals. Environmental convergence is based on investigating whether environmental policies gradually become similar over time. Firstly, Strazicich and List (2003) made a convergence analysis of CO_2_ gas with data from 1960 to 1997 in 21 industrial countries. In the following periods, EFs started to be included in convergence analysis. The EF represents sustainability and is an indicator of environmental pollution. The fact that EF represents environmental pollution more comprehensively than other environmental pollution indicators enables EF convergence to consider pollution from a wider window. The EF deals with total consumption and production instead of production and consumption activities that cause CO_2_ emissions only. The EF reveals the pressure on nature caused by all human actions, such as carbon, construction, agriculture, grassland, and fishing fields.

The convergence of pollution indicators, especially EF, can affect international climate agreements. For example, in the absence of pollution convergence, the distribution of emission permits may lead to significant migration of polluting firms (Payne 2010). Pollutant convergence is a crucial element of many climate agendas. If pollutants are not predicted to converge in the future, egalitarian environmental designs will not be successfully implemented. This is because nations with relatively low levels of emissions will be more likely to promote egalitarian agreements, as such agreements would suggest that nations with higher levels of pollution would share much of the burden of reducing pollution (Churchill et al. 2018).

## Literature review

Research on numerous environmental issues has recently piqued academics’ interest due to its vast and tremendous consequences for sustainable development. However, there is a long history of modelling environmental series’ stochastic properties. Conveniently, Solow’s (1956) unique long-run economic growth study may be used to pinpoint the origin of convergence. According to Solow (1956), if countries with an initial low level of development save and invest more, their capital accumulation would increase in comparison to the more developed countries, which would eventually lead to an economic convergence of countries’ national incomes and the disappearance of gaps in the per capita income (Bello et al. 2022). Later, due to global warming and climate change awareness, a significant number of empirical studies have been conducted in the literature that addresses the environmental convergence theory in many forms in recent years. The authors have widely employed unit root tests in the existing literature for convergence analysis. These tests can be divided into two parts. While the first part accounts for the structural breaks, the second does not. The first part, the conventional unit root test, has mainly been used in the existing literature. Since many economic series include structural breaks, tests that account for structural breaks are crucial for accurate results. In addition, the available literature uses GHG emissions, especially CO2, while some use EF. However, CO_2_ emissions represent environmental pollution on a limited basis as it only shows air pollution. On the other hand, EF is an indicator that represents environmental pollution more comprehensively but is not used by authors sufficiently. The existing literature also differs in terms of country groups.

Firstly, we classified the existing literature regarding the indicator used for environmental pollution. For this purpose, we have divided it into two-parts: the first part consists of the study investigating the convergence of CO_2_ emission, while the second part consists of the more comprehensive indicator, such as EF or its subcomponents. However, investigating the environmental pollution indicator is essential regarding the sample employed. While some studies focus on a global sample, others focus on specific regions or economic blocks. For this reason, we subdivide each part and end up with four groups of literature reviews.

The studies that employed CO_2_ for the economic or regional blocks are prevalent. Since the pioneering work of Strazicich and List (2003), which used a conventional panel unit root test to check for convergence and report significant evidence of converges in 23 industrialized countries for the period 1960–1997, the discussion on the convergence of CO_2_ emissions has been well documented in the energy and environmental economics literature. Westerlund and Basher (2008) employed conventional unit root tests for convergence in 28 industrialized countries from 1870 to 2002. They conclude that for all countries, convergence is valid. Lee et al. (2008) examined stochastic convergence in 21 OECD countries from 1960 to 2000. Using the panel seemingly unrelated regressions augmented Dickey-Fuller (SURADF) unit root test, the results provide evidence for divergence in 14 OECD countries. Barassi et al. (2008) examined the convergence of CO2 for 21 OECD countries covering the period 1950–2002 via conventional panel unit root test. The results provide no evidence of convergence. Romero-Ávila (2008) examined the existence of stochastic and deterministic convergence CO_2_ emissions in 23 countries covering the period 1960–2002 via panel stationary test. The finding provides strong evidence of both stochastic and deterministic convergence. Barassi et al. (2011) examined the convergence of CO_2_ emissions among 18 OECD members from 1870 to 2004. Utilizing a fractional integration approach, the results show evidence for fractional integration in 13 countries. Herrerias (2012) investigated CO_2_ convergence among the EU-25 countries for 2020–2007 using the distribution dynamics approach. The findings indicate that the convergence patterns differ before and after World War II, with more convergence displayed after the 1970s. El-Montasser et al. (2015) investigated the convergence hypothesis of GHG emissions among the G7 countries for the 1990–2011 period. Using a panel unit root test which provides for breaks, the results fail to find evidence for convergence in the selected countries. Jobert et al. (2010) used a Bayesian shrinkage approach to examine convergence in 22 countries, mostly OECD members, over the 1971–2006 period. The findings found evidence for absolute and conditional convergence. The results show convergence of CO_2_ in 38 countries. Yavuz and Yilanci (2013) examined the convergence of CO2 of the G7 countries for 1960–2005 using the threshold autoregressive (TAR) panel unit root test. The results showed that convergence existed in the first regime and divergence in the second. Sun et al. (2016) investigated the convergence of CO_2_ in the ten largest economies for 1971–2010, applying a stationary test with a Flexible Fourier function. The results show that CO_2_ converges in most countries. Robalino-López et al. (2016) examined the CO_2_ convergence of ten South American countries from 1980 to 2010, using the Phillips and Sul methodology. The results show that the region does not exhibit a global convergence pattern concerning CO_2_. Tiwari and Mishra (2017) investigated the convergence of CO_2_ in 18 Asian countries from 1972 to 2010, using parametric and non-parametric approaches. The results indicated convergence of all series. Finally, Presno et al. (2018) investigated the stochastic convergence in CO_2_ for 28 OECD countries covering 1901–2009 and found convergence.

The studies investigating the convergence of CO_2_ for the world sample have been less employed. Nguyen Van (2005) examined convergence in CO_2_ for 100 countries covering the period 1966–1996 and found that while convergence is valid for industrialized countries, there is little evidence of convergence for the whole sample. Aldy (2006) examined the convergence of CO_2_ in 88 countries and found evidence of converging for 23 OECD countries but showed diverging for the whole sample using the Markov chain transition matrix. Ezcurra (2007) discovered evidence of convergence across all samples using the non-parametric approach to test convergence for 87 countries from 1960 to 1999. Brock and Taylor (2010) investigated convergence in CO_2_ for 173 countries from 1960 to 1998. The finding strongly suggests convergence. Christidou et al. (2013) use unit root and stationary tests to examine the convergence of CO_2_ emissions for 36 selected countries over 1870–2006, and the results show strong evidence of convergence. Ahmed et al. (2017) examined the convergence of CO_2_ for 162 countries worldwide, covering the period 1960–2010, via wavelet-based unit root tests. Their results suggest mixed results. Using a panel of 124 countries, Runar et al. (2017) examined CO_2_ convergence between 1985 and 2010 and found that convergence occurred.

While few studies use EF as an indicator of environmental pollution in the existing literature on environmental convergence, in terms of coverage, two distinct patterns include studies focusing on a global sample of world economies and those concentrating on a specific region or economic block. Included among the studies that focused on a worldwide sample of the world’s economies, Solarin et al. (2019) tested the convergence of EF and its six components, including the footprints of built-up, carbon, cropland, fishing ground, forest land, and grazing land in 92 countries for 1961–2014. Their result provides mixed results for convergence. Haider and Akram (2019) examined the convergence of EF and CO2 for 77 countries using the Phillips and Sul technique for 1961–2014 and found no evidence of convergence. Bilgili et al. (2019), whose study of EF convergence spanned four continents, including Asia, Africa, America, and Europe, found convergence for 15 countries. Finally, Arogundade et al. (2022) examine the convergence of EF in 189 countries over 1990–2017 using a log (*t*) regression model, and the results support the evidence of convergence for the world sample.

On the other hand, some studies focus on specific regions or economic blocks. Ulucak and Apergis (2018) investigated convergence for the European Union countries covering 1961–2013. The findings provide different club convergence. Bilgili and Ulucak (2018) investigated the club convergence of the EF of G20 countries via a panel KPSS test with structural breaks, covering the period 1961–2014. The results provide evidence of convergence. Solarin (2019) examined the convergence analysis for 27 OECD countries using residual augmented least squares regression to examine the convergence of EF, carbon footprint, and CO_2_ for 1961–2013. The analysis showed that CO_2_ emissions converged in 12 countries, EF in 13 countries, and carbon footprint in 15 countries. Ulucak et al. (2020) investigated the convergence of EF and its components for sub-Saharan African countries from 1961 to 2014 using log *t* regression and found no convergence. Yilanci and Pata (2020) investigate the convergence of EF among ASEAN-5 countries using a two-regime threshold autoregressive (TAR) panel unit root test from 1961 to 2016. They found that while the first regime shows convergence, the second regime shows divergence. Erdogan and Okumus (2021) investigated a convergence analysis of EF for different country groups using a panel stationarity test with smooth shifts and log *t* methods for 1961–2016. Their results show several convergence clubs among other income groups. Işık et al. (2020) examined the convergence of EF for the UMSCA countries over the period 1961–2016 using the TAR panel unit root test. Empirical findings provide evidence of the convergence of the second regime.

The literature review above yields three significant implications within the context of the current study: (i) While other regions and economic blocks have been considered in the analysis of the convergence of EF, the BRICS-T has been conspicuously ignored, even though these country group significantly contributes to global EF. (ii) Existing literature focuses on CO_2_, an indicator representing environmental pollution in terms of air pollution, while there are several dimensions of the pollution. In the other hand, in recent times, EF has been used as a pollution indicator, which includes all dimensions of environmental pollution, but studied on a limited number. (iii) While some authors have applied the fractional integration procedure to analyze the convergence, very few previous studies have employed the method for the convergence analysis of EF despite its advantages.

## Data set and method

This study aims to examine whether the BRICS-T countries converge to the country group average. The explanations of the variables are given in Table 1.

**Table 1:** Description of variables

Variables	Definition	Period	Source
LBrazil	Logarithm of the ratio of Brazil ecological footprint to the BRICS-T average	1992–2017	Global Footprint Network
LRussia	Logarithm of the ratio of Russia ecological footprint to the BRICS-T average	1992–2017	Global Footprint Network
LIndia	Logarithm of the ratio of India ecological footprint to the BRICS-T average	1992–2017	Global Footprint Network
LChina	Logarithm of the ratio of China ecological footprint to the BRICS-T average	1992–2017	Global Footprint Network
South Africa	Logarithm of the ratio of South Africa ecological footprint to the BRICS-T average	1992–2017	Global Footprint Network
LTurkey	Logarithm of the ratio of Turkey ecological footprint to the BRICS-T average	1992–2017	Global Footprint Network

In addition, two issues were decisive in the analysis of the BRICS-T countries: (i) Assuming that BRICS-T countries have an impact on pollution at the global level because they constitute approximately 50% of the world population, they have 25% of the world GDP and cover a large geographical area. (ii) Due to the limited level of convergence in studies conducted for large country groups, convergence analysis is more widely used for classifying country groups within the framework of income, geographical region or institutional structure (Payne 2020).

Descriptive statistics for countries are given in Table 2.

**Table 2:** Descriptive statistics

	Brazil	Russia	India	China	South Africa	Turkey
Mean	2.832	5.218	0.936	2.585	3.238	2.921
Median	2.847	5.152	0.865	2.482	3.192	2.895
Maximum	3.045	6.836	1.194	3.714	3.711	3.509
Minimum	2.551	4.340	0.782	1.566	2.910	2.287
Std. dev	0.139	0.524	0.135	0.783	0.207	0.333
Skewness	−0.492	0.973	0.546	0.214	0.514	−0.183
Kurtosis	2.251	4.747	1.751	1.414	2.300	2.114
Jarque-Bera	1.655	7.411	2.982	2.9217	1.675	0.995
Probability	0.437	0.024	0.225	0.232	0.432	0.607
Observations	26	26	26	26	26	26

For convergence analysis, a data transformation was performed first. This transformation is made to the EF series at the level value of the countries. The following formula was used for this transformation (Baygın 2017; Bilgin et al. 2010; Sert and Doğan 2021).

2 $${y}_{i}^{t}=\mathrm{ln}\left({\mathrm{EF}}_{t}^{i}/{\mathrm{averageEF}}_{t}^{i}\right)$$

**Fig. 3:**
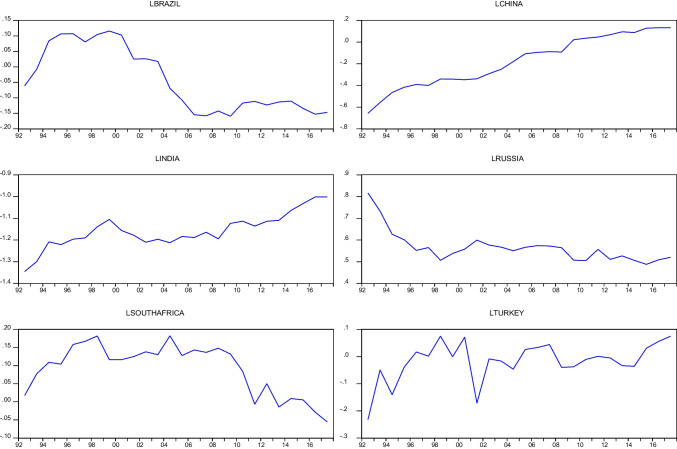
Charts of new series (${y}_{i}^{t}$) obtained for BRICS-T countries

In this equation, ${\mathrm{EF}}_{t}^{i}$ indicates the per capita EF (gha) for each country, and average ${\mathrm{EF}}_{t}^{i}$ indicates the average per capita EF of the BRICS countries. ${y}_{i}^{t}$ denotes the new series obtained for each country. In convergence analysis, unit root tests are applied to the newly obtained (${y}_{i}^{t}$) series. These series are the variables defined in Table 1. Graphics of these series are given in Fig. 3.

In this equation, ${\mathrm{EF}}_{t}^{i}$ represents the per capita EF (gha) for each country, and $\mathrm{average }{\mathrm{EF}}_{t}^{i}$ represents the average per capita EF of the BRICS countries. ${y}_{i}^{t}$ denotes the new series obtained for each country. In convergence analysis, unit root tests are applied to the newly obtained (${y}_{i}^{t}$) series. The graphs of the new series obtained for the BRICS-T countries are shown in Fig. 3.

In the unit root tests used in this study, the basic hypothesis shows that the series has a unit root. That is, there is no convergence. At the same time, the alternative theory states that the series is stationary, that is, there is convergence. If the calculated test value is less than the table critical value, the H_0_ hypothesis is rejected. That is, the series is said to converge.

## Empirical methodology

This study used the Fourier ADF unit root test developed by Enders and Lee (2012) and the Fractional Fourier unit root tests developed by Bozoklu et al. (2020). Unit root tests with the Fourier approach have some advantages over other tests. Ignoring the structural breaks in the series may lead to the non-rejection of the unit root hypothesis in the case of structural breaks (Perron 1989). Most unit root tests that consider the structural changes in the literature allow structural breaks through dummy variables. On the other hand, dummy variables only capture the dynamics of sudden changes (Bozoklu et al. 2020). The Fourier approach, on the other hand, can capture the structural changes of unknown functions. The location, number, and form of structural changes do not affect the power of the test (Becker et al. 2006). The advantages of the Fourier approach have been instrumental in using these models for convergence analysis.

In their study, Enders and Lee (2012) extended the ADF unit root test with a Fourier function. In the study, it has been shown that one or more structural changes can be predicted with low-Frequency Fourier functions. It is not necessary to know the number and location of the structural change in the test application. The appropriate frequency value of the Fourier function needs to be estimated. In the first stage of the test, the following model is estimated.

3 $$\Delta {y}_{t}=\rho {y}_{t-1}+{c}_{1}+{c}_{2}\mathrm{trend}+{c}_{3}\mathrm{sin}\left(\frac{2\pi kt}{T}\right)+{c}_{4}\mathrm{cos}\left(\frac{2\pi kt}{T}\right)+{u}_{t}$$

In this equation, *t* represents the trend, *T* represents the number of observations, $\mathrm{sin}\left(\frac{2\pi kt}{T}\right)\mathrm{and} \mathrm{cos}\left(\frac{2\pi kt}{T}\right)$ represent the trigonometric terms (Fourier function), respectively.

In the first step, in order to find the optimal value of *k*, 1, 2, 3, 4, and 5 values are written instead of *k*, and the model is estimated. The *k* giving the smallest SSR is said to be suitable. If there is an autocorrelation problem in the model with the appropriate *k* value, the lagged dependent variable is included in the model, and the model is estimated. In order to use this test, a pretest should be done. The diagnostic tests the significance of the Fourier functions *c*_3_ and *c*_4_. To test the significance of trigonometric terms:

$${c}_{3}={c}_{4}=0,$$

the hypothesis is established, and testing is carried out with the *F* test. The calculated *F* statistic, *F*(*k* ^), is compared with the sample size in Tables 1a and   1b in the article of Enders and Lee (2012) and also compared with the critical values of $F\left(\widehat{k}\right)=$ Max $F(k)$ whether the model contains a trend or not.

If the observation value of *F* is less than the critical value, the H_0_ hypothesis is not rejected. If the trigonometric terms are significant, the Fourier function is significant. In this case, the Fourier ADF unit root test can be used. Otherwise, the ADF unit root test should be used. If the Fourier function is statistically significant, ${y}_{t-1}$ significance is tested in the model to test the basic hypothesis that there is a unit root. Appropriate critical values are found in the article by Enders and Lee (2012). If the calculated test statistic is less than the table critical value, the basic hypothesis is rejected, and the series is said to be stationary.

Christopoulos and Leon-Ledesma (2011) showed in their study that the frequency value (*k*) in the Fourier function could be fractional numbers. Omay (2015) developed the frequency values in the study of Enders and Lee (2012), who developed the Fourier ADF unit root test, to be fractional. In the study, it has been shown that the *k* value can take values from 0 to a maximum value of 2 with 0.1 increments. It is stated that setting the increment value as 0.1 makes it possible to limit excessive filtering and non-linear trend problems. Bozoklu et al. (2020), on the other hand, removed the restriction of frequency values to be between 0 and 2 and allowed this frequency to have fractional values between 0 and 5. According to the appropriate frequency values, the critical values for the unit root hypothesis were obtained by Monte Carlo simulation and tabulated in the article. Thus, they brought the Fractional Fourier ADF unit root test to the literature.

The following model is estimated to apply the Fourier ADF unit root test.

4 $$\Delta {y}_{t}={\delta }_{0}+{\delta }_{1}\mathrm{sin}\left(\frac{2\pi kt}{T}\right)+{\delta }_{2}\mathrm{cos}\left(\frac{2\pi kt}{T}\right)+{\delta }_{3}{y}_{t-1}+\sum_{i=1}^{p}{\alpha }_{i}\Delta {y}_{t-i}+{v}_{t}$$

In this equation, *t* represents the trend term, *T* is the number of observations, *π* = 3.1416, *k* is the specific frequency, $\mathrm{sin}\left(\frac{2\pi kt}{T}\right)\mathrm{ve cos}\left(\frac{2\pi kt}{T}\right)\mathrm{ is}$ the trigonometric terms (Fourier function), and *p* represents the optimal lag length. Akaike information criterion was used to determine the optimal lag length.

In order to find the appropriate value of* k*, values in the form of [0.1, 0.2,…,5] are put in place of *k*, increasing by 0.1 units. The *k* value that gives the smallest SSR is determined as the appropriate *k* value. To test the significance of trigonometric terms, the hypothesis ${\delta }_{1}={\delta }_{1}=0$ is tested. When deciding on this test, appropriate critical values are obtained from the article by Enders and Lee (2012).

Since the ADF unit root test cannot account for structural breaks, it can give biased results. Because many economic series contain structural breaks in the long run, the Fourier ADF unit root test developed by Enders and Lee (2012) considers structural breaks. However, this test does not consider fractional values in determining the optimum lag length. In the Fractional Frequency Fourier ADF test developed by Bozoklu et al. (2020), fractional values can be given in determining the *k* (Bozoklu et al. 2020). In the determination of *k*, approximately 50 models are estimated between 0.1 and 5, and the *k* value that minimizes the mean squared error is determined as the optimum *k* value. Therefore, the test is likely to give more accurate results in determining the stationarity of the series.

## Empirical findings

According to the ADF unit root test result presented in Table 3, the per capita EF converges to the BRICS-T average at a 1% significance level for Russia and Turkey. On the other hand, there is no convergence for Brazil, India, China, and South Africa.

**Table 3:** ADF unit root test results

Variables	Test statistic
LBrazil	−2.8007 (0)
LRussia	−4.6306^a^ (0)
LIndia	−2.3162 (0)
LChina	−2.8490 (0)
LSouth Africa	−2.6132 (0)
LTurkey	−5.3077^a^ (0)

^a^Indicates that the H_0_ unit root hypothesis is rejected at the 1% significance level. Values in parentheses indicate appropriate lag lengths, and the maximum lag length is taken as 3. Schwarz information criterion was used to determine the optimal lag length

The results of the Fourier ADF unit root test developed by Enders and Lee (2012) are given in Table 4.

**Table 4:** Fourier ADF unit root test results

Countries	Frequency (k)	Min SSR	F test statistics $F(\widehat{k})$	Optimal lag	FADF test statistics
Brazil	1	0.0146	2.0407	6	−2.0233
Russia	5	0.0128	19.759^a^	4	−4.1965**
India	1	0.0138	8.5846^c^	6	−3.8950
China	1	0.0129	11.163^b^	5	−4.6918**
South Africa	1	0.0184	9.5443^b^	6	−2.2750
Turkey	3	0.0499	5.0013	6	−3.7816

^a^, ^b^, and ^c^ show that the trigonometric terms are statistically significant at the 1%, 5%, and 10% significance levels, respectively. **Indicates that the series is stationary at the 5% significance level. The values in the table are obtained for the fixed and trend model

In Table 4, trigonometric terms were found to be statistically significant for Russia, India, China, and South Africa. Therefore, the Fourier function is significant for these countries and this reason. The Fourier ADF unit root test can be used. However, trigonometric terms were not statistically significant for Brazil and Turkey. Therefore, the Fourier ADF unit root test cannot be used for these countries. ADF test can be used for Brazil and Turkey. Since the FADF test statistics for Russia and China were smaller than the table critical value at the 5% significance level, the series was stationary at the 5% significance level. In other words, Russia and China’s per capita EF converge to the BRICS-T average, and there is no convergence for South Africa and India. Since the trigonometric terms are not significant for Brazil and Turkey, the ADF unit root test results are valid instead of the Fourier ADF unit root test for convergence analysis for these countries.

The results of the Fractional Fourier ADF unit root test developed by Bozoklu et al. (2020) are given in Table 5.

**Table 5:** Fractional Frequency Fourier ADF unit root test results

Countries	Frequency (k)	Min SSR	*F* test statistics $F(\widehat{k})$	Optimal lag	FADF test statistics
Brazil	1.6	0.0102	17.3932^a^	1	−5.9511***
Russia	4.5	0.0126	1.8809	6	−3.8395
India	1.3	0.0131	7.2167	6	−3.8576
China	1.5	0.0124	11.468^b^	5	−4.5371**
South Africa	0.8	0.0182	9.2335^b^	6	−2.2914
Turkey	2.8	0.0495	2.9385	6	−3.0026

^a^, ^b^, and ^c^ show that the trigonometric terms are statistically significant at the 1%, 5% and 10% significance levels, respectively. *** and * indicate that the series are stationary at the 1% and 10% significance levels, respectively. The values in the table are obtained for the fixed and trend model

The fact that the $F(\widehat{k})$ statistic in the fourth column of Table 5 is significant and indicates that trigonometric values should be added to all estimation models. Trigonometric terms were significant for Brazil, Russia, and South Africa. Therefore, the Fourier function is statistically significant. Therefore, the Fractional Fourier ADF unit root test can be used for Brazil, China, and South Africa. For other countries, the ADF unit root test results are valid. When the FADF test statistic calculated for Brazil (Bozoklu et al. 2020) is compared with the critical table values, the basic hypothesis stating that the series has a unit root at the 1% significance level is rejected. In other words, Brazil’s average per capita EF converges with the BRICS-T average. The FADF test statistic calculated for China was found to be stationary at the 5% significance level. Therefore, China’s per capita EF converges with the BRICS-T average. Although South Africa’s Fourier function is significant, the unit root hypothesis cannot be rejected. That is, there is no convergence in the series. Since trigonometric terms are not significant according to Fractional Fourier ADF unit root test results, ADF unit root test results are valid for Russia, India, and Turkey.

## Conclusion and recommendations

Environmental issues such as pollution and climate change have been frequent discussion topics recently. Testing pollution convergence through unit root tests has recently been an exciting topic for policymakers, as environmental indicators provide information on environmental protection policies. The existence of convergence reveals that the effects of policies will be temporary; otherwise, impacts will be permanent. From this point on, our study can provide policymakers with information on the impact of policies.

The existing literature on environmental degradation generally analyzes the stationarity properties of CO_2_ emissions to evaluate whether policies will be in effect. However, environmental pollution cannot be defined by air pollution alone, and other parts of environmental degradation include the degradation of soil, forestry, mining, and oil. In addition, CO_2_ emissions may decrease due to technological innovations or strict environmental regulations by governments, while overall waste and pollution levels increase (Stern 2014). Thus, the results may be misleading if policy recommendations are made using only CO_2_ as the total environmental impact variable. Because the carbon footprint component of the EF contains human-caused CO_2_ emissions, it represents the world’s need to transition from using fossil fuels (Ulucak and Lin 2017). For this reason, it is essential to evaluate environmental pollution through EF, which performs environmental pollution more comprehensively, to implement more practical policies.

Existing literature on the convergence of environmental pollution is divided into two parts. The first part uses gas emissions as a pollution indicator, while the latter uses more comprehensive indicators, such as EF representing environmental degradation rather than pollution. The pioneering work of Strazicich and List (2003) on the convergence analysis of CO_2_ plays a significant role in the inclusion of environmental pollution variables in the convergence analysis. However, the empirical literature on this subject is mainly based on studies using indicators that partially reflect environmental pollution. On the other hand, EF has been used in the convergence literature in recent years as a more comprehensive and effective indicator of environmental degradation. However, it has not been used enough.

In this study, we investigate whether the per capita EF of BRICS-T members converges to the BRICS-T average over the period 1992–2017. Since studies on the convergence of EF is scanty compared to the numerous studies on the convergence of CO_2_, we use per capita EF to reflect environmental pollution, more precisely, environmental degradation. The BRICS-T countries have an essential position in terms of the global economy, population, and land area, which are the main factors that increase the EF. China, India, and Russia have a large share of the world’s exports. As export is not included in the calculation of the exporter countries’ EF but in the importer countries’, analyzing the convergence of these countries is crucial not only for the BRICS-T but for the world in terms of pollution mitigation.

Moreover, to the best of our knowledge, no study has examined BRICS-T convergence, while studies have generally used some other regional or economic blocks. Finally, in addition to the conventional ADF unit root test, we use the Fourier ADF unit root test developed by Enders and Lee (2012), which considers structural breaks, and the Fractional Fourier Frequency ADF unit root test developed by Bozoklu et al. (2020), which considers fractional values to account for structural breaks. Given this information, our study fills the gap in the existing literature, particularly regarding methodology, indicator, and sample.

Our results showed that EF converges in Russia and Turkey according to the conventional ADF test, in China and Russia according to the Fourier ADF test, and in Brazil and China according to the Fractional Fourier Frequency test. Since the Fractional Fourier Frequency ADF test is more powerful in getting accurate results, we found that EF converges in China and Brazil but does not converge in Russia, India, Turkey, and South Africa briefly. The lack of convergence on an environmental indicator is likely due to the limited degree of economic, social, and political integration among the region’s countries. Working together to address environmental challenges can become more credible and effective in case of convergence. Given that Brazil’s and China’s EFs are converging, this suggests that these countries’ policies or the shocks are temporary in the long run. China has the highest GDP after the USA and is the most populous country, and its production depends on foreign energy. Therefore, any policy that lessens EF helps the world’s environmental degradation abatement. As the results show convergence, radical and proactive measures will be more effective than common policies. In this context, a strict tax policy on the actions and products that harm the environment, using clean and renewable energy, and lessening fossil fuel usage, can reduce pollution.

Similarly, as Brazil’s EF is converging, proactive policies could contribute to a reduction in pollution. On the other hand, since no evidence was found for convergence, the effects of the common policies will be permanent in India, Russia, Turkey, and South Africa. However, this does not mean that these countries are successful in their efforts to reduce pollution as the EF continues to rise.

The study can serve as a guide for future studies. For example, making separate convergence analyses for the EF components of these countries’ grassland, agricultural, forest, construction, and carbon footprint variables can explain which policies could be prioritized for which footprint. In addition, the impact of Covid-19 can be observed after the publication of the new data by the Global Footprint Network.

## Data Availability

All data generated or analyzed during this study are included in this published article.

## References

[CR1] Ahmed M, Khan AM, Bibi S, Zakaria M (2017). Convergence of per capita CO2 emissions across the globe: insights via wavelet analysis. Renew Sustain Energy Rev.

[CR2] Aldy JE (2006). Per capita carbon dioxide emissions: convergence or divergence?. Environ Resource Econ.

[CR3] Arogundade S, Hassan A, Akpa E, Mduduzi B (2023) Closer together or farther apart: are there club convergence in ecological foot-print? Environ Sci Pollut Res 30(6):15293–15310. 10.1007/s11356-022-23203-5 10.1007/s11356-022-23203-5 36169845

[CR4] Balsalobre-Lorente D, Driha OM, Halkos G, Mishra S (2022). Influence of growth and urbanization on CO2 emissions: the moderating effect of foreign direct investment on energy use in BRICS. Sustain Dev.

[CR5] Barassi MR, Cole MA, Elliott RJR (2008). Stochastic divergence or convergence of per capita carbon dioxide emissions: re-examining the evidence. Environ Resource Econ.

[CR6] Barassi MR, Cole MA, Elliott RJR (2011). The stochastic convergence of CO2 emissions: a long memory approach. Environ Resource Econ.

[CR7] Barro RJ, Sala-i-Martin X (1992). Convergence. J Political Econ.

[CR8] Baygın BK (2017) Stochastic convergence of per capita greenhouse gas emissions among G7 countries. İstanbul Üniversitesi İktisat Fakültesi Ekonometri ve İstatistik Dergisi 26:60–70

[CR9] Becker R, Enders W, Lee J (2006). A stationarity test in the presence of an unknown number of smooth breaks. J Time Series Analysis.

[CR10] Bello MO, Gil-Alana LA, Ch’ng GS (2022). Mean reversion and convergence of ecological footprint in the MENA region: evidence from a fractional integration procedure. Environ Sci Pollut Res.

[CR11] Bilgili F, Ulucak R (2018) Is there deterministic, stochastic, and/or club convergence in ecological footprint indicator among G20 countries? Environ Sci Pollut Res 25:35404–35419. 10.1007/s11356-018-3457-1 10.1007/s11356-018-3457-1 30350136

[CR12] Bilgili F, Ulucak R, Koçak E (2019) Implications of environmental convergence: continental evidence based on ecological foot-print. Energy and environmental strategies in the era of globali- zation, Springer, Berlin, pp 133–165

[CR13] Bilgin MH, Lau CKM, Demir E, Astrauskiene N (2010). Rental price convergence in a developing economy: new evidence from non-linear panel unit root test. Int J Strateg Prop Manag.

[CR14] Borucke M, Moore D, Cranston G, Gracey K, Iha K, Larson J, Lazarus E, Morales JC, Wackernagel M, Galli A (2013). Accounting for demand and supply of the biosphere’s regenerative capacity: the National Footprint Accounts’ underlying methodology and framework. Ecol Ind.

[CR15] Bozoklu Ş, Yilanci V, Gorus MS (2020) Persistence in per capita energy consumption: a fractional integration approach with a Fourier function. Energy Econ 91:104926. 10.1016/j.eneco.2020.104926

[CR16] Brock WA, Taylor MS (2003) The kindergarten rule of sustainable growth. Massachutes (Working Paper 9597)

[CR17] Brock WA, Taylor MS (2010). The Green Solow model. J Econ Growth.

[CR18] Burnett JW (2016). Club convergence and clustering of U.S. energy-related CO 2 emissions. Resour Energy Econ.

[CR19] Christidou M, Panagiotidis T, Sharma A (2013). On the stationarity of per capita carbon dioxide emissions over a century. Econ Model.

[CR20] Christopoulos DK, Leon-Ledesma MA (2011) International output convergence, breaks, and asymmetric adjustment. Stud Nonlinear Dyn Econom 15(3):1558–3708

[CR21] Churchill SA, Inekwe J, Ivanovski K (2018). Conditional convergence in per capita carbon emissions since 1900. Appl Energy.

[CR22] Diallo IA (2004) The environmental Kuznets curve in a public spending model of economic growth, MPRA. https://mpra.ub.uni-muenchen.de/56528/

[CR23] El-Montasser G, Inglesi-Lotz R, Gupta R (2015). Convergence of greenhouse gas emissions among G7 countries. Appl Econ.

[CR24] Enders W, Lee J (2012). The flexible Fourier form and Dickey-Fuller type unit root tests. Econ Lett.

[CR25] Erdogan S, Okumus I (2021). Stochastic and club convergence of EF: an empirical analysis for different income group of countries. Ecol Ind.

[CR26] Ewing B, Moore D, Goldfinger S, Oursler A, Reed A, Wackernagel M (2010) EF Atlas 2010. GLOBAL FOOTPRINT NETWORK

[CR27] Ezcurra R (2007). Is there cross-country convergence in carbon dioxide emissions?. Energy Policy.

[CR28] Galli A, Kitzes J, Wermer P, Wackernagel M, Niccolucci V, Tiezzi E (2007). An exploration of the mathematics behind the EF. Int J Ecodyn.

[CR29] Galli A, Wackernagel M, Iha K, Lazarus E (2014). EF: implications for biodiversity. Biol Cons.

[CR30] Galor O (1996). Convergence? Inferences from theoretical models. Econ J.

[CR31] Grossman GM, Krueger AB (1991) Environmental impacts of a North American free trade agreement, NBER Working Paper Series, Working Paper No: 3914, NBER

[CR32] Haider S, Akram V (2019). Club convergence analysis of ecological and carbon footprint: evidence from a cross-country analysis. Carbon Manag.

[CR33] Herrerias MJ (2012). CO2 weighted convergence across the EU-25 countries (1920–2007). Appl Energy.

[CR34] Işık C, Sirakaya-Turk E, Ongan S (2020) Testing the efficacy of the economic policy uncertainty index on tourism demand in USMCA: Theory and evidence. Tour Econ 26(8):1344–1357

[CR35] Jobert T, Karanfil F, Tykhonenko A (2010). Convergence of per capita carbon dioxide emissions in the EU: legend or reality?. Energy Econ.

[CR36] Kitzes J, Peller A, Goldfinger S, Wackernagel M (2007) Current methods for calculating national ecological footprint accounts. Sci Environ Sustain Soc 4(1):1–9

[CR37] Kitzes J, Galli A, Bagliani M, Barrett J, Dige G, Ede S, Erb K, Giljum S, Haberl H, Hails C, Jolia-Ferrier L, Jungwirth S, Lenzen M, Lewis K, Loh J, Marchettini N, Messinger H, Milne K, Moles R, Wiedmann T (2009). A research agenda for improving national EF accounts. Ecol Econ.

[CR38] Kuznets S (1955). Economic growth and income inequality. Am Econ Rev.

[CR39] Lee C-C, Chang C-P, Chen P-F (2008). Do CO2emission levels converge among 21 OECD countries? New evidence from unit root structural break tests. Appl Econ Lett.

[CR40] Lin D, Hanscom L, Martindill J, Borucke M, Cohen L, Galli A, Lazarus E, Zokai G, Wackernagel KIM (2018) Working Guidebook to the National Footprint Accounts. Global Footprint Network

[CR41] Ma X, Jiao S (2023). Comprehensive analysis of water resources from the perspective of water footprint and water ecological footprint: a case study from Anyang City, China. Environ Sci Pollut Res.

[CR42] Mathis W, David YJ (1998). The EF an indicator of progress toward regional sustainability. Environ Monit Assess.

[CR43] Monfreda C, Wackernagel M, Deumling D (2004). Establishing national natural capital accounts based on detailed EF and biological capacity assessments. Land Use Policy.

[CR44] Van Nguyen P (2005). Distribution dynamics of CO2 emissions. Environ Resource Econ.

[CR45] Omay T (2015) Fractional frequency flexible Fourier form to approximate smooth breaks in unit root testing. Econ Lett 134:123–126. 10.1016/j.econlet.2015.07.010

[CR46] Ozcan B, Ulucak R, Dogan E (2019). Analyzing long lasting effects of environmental policies: evidence from low, middle and high income economies. Sustain Cities Soc.

[CR47] Payne JE (2010). A survey of the electricity consumption-growth lit- erature. Appl Energy.

[CR48] Payne JE (2020). The convergence of carbon dioxide emissions: a survey of the empirical literature. J Econ Stud.

[CR49] Perron P (1989). The great crash, the oil price shock, and the unit root hypothesis. Econometrica.

[CR50] Pradhan P, Costa L, Rybski D, Lucht W, Kropp JP (2017). A systematic study of sustainable development goal (SDG) interactions. Earth’s Future.

[CR51] Presno MJ, Landajo M, Fernández González P (2018). Stochastic convergence in per capita CO 2 emissions. An approach from non-linear stationarity analysis. Energy Econ.

[CR52] Robalino-López A, García-Ramos JE, Golpe AA, Mena-Nieto A (2016). CO2 emissions convergence among 10 South American countries. A study of Kaya components (1980–2010). Carbon Manag.

[CR53] Romero-Ávila D (2008). Convergence in carbon dioxide emissions among industrialized countries revisited. Energy Econ.

[CR54] Runar B, Amin K, Patrik S (2017). Convergence in carbon dioxide emissions and the role of growth and institutions: a parametric and non-parametric analysis. Environ Econ Policy Stud.

[CR55] Sert F, Doğan S (2021) Enerji Yoğunluğu ve İklim Değişikliği: Türkiye İçin Yakınsama Analizi. Ekoist: J Econ Stat 32:1–14. 10.26650/ekoist.2020.32.0099

[CR56] Solarin SA (2019). Convergence in CO 2 emissions, carbon footprint and EF: evidence from OECD countries. Environ Sci Pollut Res Int.

[CR57] Solarin SA, Tiwari AK, Bello MO (2019) A multi-country convergence analysis of EF and its components. Sustain Cities Soc 46:101422. 10.1016/j.scs.2019.101422

[CR58] Solow RM (1956). A contribution to the theory of economic growth. Q J Econ.

[CR59] Stern DI (2014) The environmental Kuznets curve: a primer (CCEP working paper 1404)

[CR60] Stern DI (2017). The environmental Kuznets curve after 25 years. J Bioecon.

[CR61] Strazicich MC, List JA (2003). Are CO2 emission levels converging among industrial countries?. Environ Resource Econ.

[CR62] Sun J, Su C-W, Shao G-L (2016). Is carbon dioxide emission convergence in the ten largest economies?. Int J Green Energy.

[CR63] Tiwari C, Mishra M (2017). Testing the CO2 emissions convergence: evidence from Asian countries. IIM Kozhikode Society & Management Review.

[CR64] Ulucak R, Lin D (2017) Persistence of policy shocks to ecological footprint of the USA. Ecol Indic 80:337–343

[CR65] Ulucak R, Bilgili F (2018). A reinvestigation of EKC model by ecological footprint measurement for high, middle and low income countries. J Clean Prod.

[CR66] Ulucak R, Apergis N (2018). Does convergence really matter for the environment? An application based on club convergence and on the ecological footprint concept for the EU countries. Environ Sci Policy.

[CR67] Ulucak R, Kassouri Y, Çağrı İlkay S, Altıntaş H, Garang APM (2020). Does convergence contribute to reshaping sustainable development policies? Insights from Sub-Saharan Africa. Ecol Indic.

[CR68] Wackernagel M, Schulz NB, Deumling D, Linares AC, Jenkins M, Kapos V, Monfreda C, Loh J, Myers N, Norgaard R, Randers J (2002) Tracking the ecological overshoot of the human economy. Proc Natl Acad Sci USA 99(14):9266–9271 10.1073/pnas.142033699 PMC123129 12089326

[CR69] Westerlund J, Basher SA (2008). Testing for convergence in carbon dioxide emissions using a century of panel data. Environ Resource Econ.

[CR70] Williams III RC (2016) Environmental taxation. NBER Working Paper, Massasuthes (Working Paper 22303)

[CR71] Wright LA, Kemp S, Williams I (2011). Carbon footprinting: towards a universally accepted definition. Carbon Manag.

[CR72] Yavuz NC, Yilanci V (2013). Convergence in per capita carbon dioxide emissions among G7 countries: a TAR panel unit root approach. Environ Resource Econ.

[CR73] Yilanci V, Pata UK (2020) Convergence of per capita EF among the ASEAN-5 countries: evidence from a non-linear panel unit root test. Ecol Indic 113:106178. 10.1016/j.ecolind.2020.106178

